# Long-term retrieval performance is associated with CA1 hippocampal volume in older adults and individuals at risk for dementia

**DOI:** 10.1186/s13195-025-01833-4

**Published:** 2025-08-22

**Authors:** Claudia Bartels, Joy Tzu-Yueh Chen, Michael Belz, Renat Yakupov, Emrah Düzel, Wenzel Glanz, Falk Lüsebrink, Peter Dechent, Luca Kleineidam, Melina Stark, Annika Spottke, Marie Coenjaerts, Klaus Fließbach, Anja Schneider, Ayda Rostamzadeh, Frank Jessen, Björn H. Schott, Jens Wiltfang, Ingo Frommann, Michael Wagner, Roberto Goya-Maldonado

**Affiliations:** 1 Department of Psychiatry and Psychotherapy, University Medical Center Goettingen, Von-Siebold-Str. 5, D-37075 Goettingen, Germany; 2 Laboratory of Systems Neuroscience and Imaging in Psychiatry (SNIP-Lab), Department of Psychiatry and Psychotherapy, University Medical Center Goettingen, Goettingen, Germany; 3 German Center for Neurodegenerative Diseases (DZNE), Magdeburg, Germany; 4 Institute of Cognitive Neurology and Dementia Research (IKND), Otto-von-Guericke University, Magdeburg, Germany; 5 MR-Research in Neurosciences, Department of Cognitive Neurology, Georg-August- University Goettingen, Goettingen, Germany; 6 German Center for Neurodegenerative Diseases (DZNE), Bonn, Germany; 7 Department of Old Age Psychiatry and Cognitive Disorders, University Hospital Bonn, University of Bonn, Bonn, Germany; 8 Department of Neurology, University Hospital Bonn and University of Bonn, Bonn, Germany; 9 Department of Psychiatry, Medical Faculty, University of Cologne, Cologne, Germany; 10 Excellence Cluster on Cellular Stress Responses in Aging-Associated Diseases (CECAD), University of Cologne, Cologne, Germany; 11 German Center for Neurodegenerative Diseases (DZNE), Goettingen, Germany; 12 Leibniz Institute for Neurobiology, Magdeburg, Germany; 13 Neurosciences and Signaling Group, Institute of Biomedicine (iBiMED), Department of Medical Sciences, University of Aveiro, Aveiro, Portugal

**Keywords:** Long-term retrieval, Memory, Consolidation, Hippocampus, Subjective cognitive decline, Mild cognitive impairment, Dementia, Alzheimer’s disease

## Abstract

**Background:**

Long-term retrieval (LTR) and accelerated long-term forgetting (ALF) paradigms might help differentiating individuals at increased dementia risk from healthy controls (HC).

**Objective:**

We investigated the utility of a LTR paradigm in discriminating subjective cognitive decline (SCD) from HC and its relationship to the CA1 body volume, a hippocampal structure pivotal to the memory circuitry.

**Methods:**

LTR was assessed via recall rates of the ADAS-cog word list and the FCSRT-IR free recall in 59 DELCODE study participants, including individuals with SCD and mild cognitive impairment (MCI), as well as HC, all of them DELCODE study participants. LTR performance was compared between groups and its discriminability between SCD and HC was assessed using ROC curve analysis. 32 SCD and HC participants had FreeSurfer-segmented MRI data, and hippocampal subfield volumes were correlated with LTR rates.

**Results:**

Only FCSRT-IR LTR rates sufficiently differentiated SCD from HC (AUC of 0.701; 95% CI 0.537–0.865). Moderate associations of the FCSRT-IR LTR rate with CA1 bodies in both hemispheres (left CA1 body *r* = 0.419, *p* = 0.017; right: *r* = 0.412, *p* = 0.019), in addition to the left C3 body were observed (*r* = 0.525, *p* = 0.002).

**Conclusions:**

LTR may constitute a potential indicator of memory circuitry integrity in older adults, which is also mirrored by its association with CA1 volume. Thus, assessment of LTR and associated neural circuits may help to better identify individuals at risk for future cognitive decline today indistinguishable from HC, ultimately paving the way for early intervention.

**Supplementary Information:**

The online version contains supplementary material available at 10.1186/s13195-025-01833-4.

## Introduction

Subjective cognitive decline (SCD) has been defined as a subjectively perceived impairment of cognitive function unrelated to an acute event and is often accompanied by worries on cognitive decline [1, 2]. SCD has attracted increasing attention in translational and clinical research as it has been identified as a preclinical risk state for dementia which precedes measurable cognitive loss or includes only subtle changes. Therefore, SCD is typically not detectable by standard neuropsychological procedures [1–3]. Specifically, subjective memory loss and related concerns are mainly associated with progression to amnestic mild cognitive impairment (MCI) and Alzheimer’s disease (AD) dementia [4]. These subtle cognitive changes, including subjective experiences of cognitive decline, occur at late stages of the preclinical, cognitively unimpaired phase in the AD continuum (preclinical AD stage 2) [5], and thus precede the more pronounced cognitive impairments measurable with standard neuropsychological tests comprising delayed recall of information with retrieval intervals up to one hour.

Substantially prolonging retrieval intervals may represent one approach to improving the detection of subtle changes in SCD, by increasing the demands of explicit/episodic memory tasks (e.g., [6, 7]). Assessing long-term memory consolidation through long-term retrieval (LTR) after several days to months may constitute a promising paradigm for identifying such subtle cognitive changes. Long-term consolidation refers to the process of stabilizing newly learned and still fragile information by integrating it into existing memory networks [8]. Closely linked to long-term consolidation is LTR, defined as the process of reaccessing this information. Over time, memory typically decays [9]; however, in some conditions, abnormal forgetting may occur. This higher rate of forgetting over time refers to the phenomenon of accelerated long-term forgetting (ALF). ALF as such describes a process in which information that has been successfully (over)learned, encoded, and retained for up to one hour is subsequently forgotten more rapidly over hours, days, or weeks [10, 11]. ALF could represent impairments in both consolidation and retrieval of long-term memory (i.e., LTR [10]), and thus precede explicit memory impairment as measured with standard tests.

LTR– or, seen from a deficit perspective with initial successful encoding, termed as ALF– has already been investigated in other neurological conditions associated with medial temporal lobe (MTL) damage (especially temporal lobe epilepsy (TLE), e.g., [12–15]). Importantly, ALF does not seem to be an epilepsy-specific or seizure-related phenomenon, but has also been observed in children who have sustained severe traumatic brain injury and diffuse subcortical damage [16]. Using different paradigms, ALF was also found in patients with AD dementia [17]. With particular relevance for a potential utility in early diagnosis, ALF was also found in preclinical stages of AD. Already in 2008, a small study in older adults with memory complaints but normal cognitive performance in standard tests (a definition compatible with the later term SCD, *n* = 10) revealed impairments in verbal and visual memory recall six weeks after initial learning in comparison to controls (*n* = 9) [18]. In more recent years, an explorative study reported higher long-term forgetting rates after three months in cognitively healthy individuals with pathological CSF AD biomarkers (preclinical AD stage 1, *n* = 14) compared to 31 controls [19]. A subgroup analysis also found ALF after three months in *n* = 11 cognitively asymptomatic ApoE4 carriers when compared to *n* = 11 sex-, age-, and education-matched noncarriers [20]. The same author group also investigated ALF in SCD patients with high scores in the SCD questionnaire (“high SCD”, *n* = 31) compared to “low SCD” (*n* = 21) and showed a significantly higher forgetting rate for high SCD at three months. ALF was furthermore more pronounced when stratified by amyloid status [21]. ALF was also found to be a feature of presymptomatic autosomal dominant (familial) AD with *n* = 21 APP or PSEN1 mutation carriers recalling smaller proportions of learned material seven days later compared to *n* = 14 noncarriers [22]. An event-based modelling approach to estimate the sequence of cognitive decline in this condition revealed that preclinical cognitive change in mutation carriers became detectable first in ALF measures study [23]. For an overview of ALF in neurodegenerative diseases, please see also [11]. A very recent study added up to this evidence and showed higher ALF rates 24 h and seven days later in *n* = 13 individuals with severe SCD in comparison to *n* = 16 healthy controls. An additional, explorative magnetic resonance imaging (MRI) analysis of the whole SCD sample (mild and severe SCD) displayed associations between functional connectivity values within some cortical networks involved in memory [24]. Moreover, a previous study in cognitively healthy older adults suggests that a combination of ALF and hippocampal volumetric measures may constitute a better predictor of one-year cognitive deterioration than standard neuropsychological tests alone [25]. However, the hippocampus comprises different subfields that show a certain degree of functional specialization with respect to distinct subprocesses of memory function [26–29].

ALF as a deficit in LTR might indicate an early dysfunction in hippocampus-dependent circuits, particularly in the presence of AD-related pathology like amyloid and Tau deposition, but little is known about the overt impact on key structures behind intermediate- and long-term memory in this context. This issue was addressed in a recent study that investigated the relationship between ALF and the hippocampal subfield cornu ammonis (CA) 1 in patients with focal epilepsy [30]. The volume of the CA1 body, a region previously implicated in memory encoding and retrieval circuit in clinical [30, 31] and animal studies [32], has been associated with ALF in a verbal memory task. This opens a new perspective for investigating the CA1 volume in relation to ALF in SCD, with the potential to identify an objective measure in a risk group for developing AD dementia that has previously only been characterized by subjective measures.

In this study, we investigated MCI, SCD, and healthy controls (HC), participants from the multicenter DELCODE study, which encompasses a comprehensive neuropsychological assessment and automated, quality-checked hippocampal subfield segmentations from MRI data. LTR data were acquired from a subsample, constituting the data base of the present study. Based on the current evidence, we hypothesized that LTR rates (1) could be used to discriminate SCD and HC participants, and (2) would be associated with CA1 body volumes.

## Materials and methods

### DELCODE study

Data origins from the DELCODE (DZNE Longitudinal Cognitive Impairment and Dementia) Study, an observational longitudinal multicenter study on deep phenotyping and longitudinal assessment of predementia at-risk states of AD run by the German Center for Neurodegenerative Diseases (DZNE). Study protocol details have been described in detail elsewhere [33]. Briefly, ten university-based DZNE partner memory clinics in Germany enrolled cognitively healthy controls (HC), individuals with SCD, MCI, and mild AD dementia, as well as first-degree relatives of AD dementia patients. Participants underwent annual assessments, comprising comprehensive clinical and neuropsychological testing, MRI, sampling of blood, urine and, in a subsample, cerebrospinal fluid (CSF). Major eligibility criteria were the following: (1) age of ≥ 60 years, (2) fluent German language skills, (3) capacity to provide written informed consent, and (4) availability of a study partner. Individuals with SCD, MCI and AD dementia were (self-)referrals to the memory clinics, while HC and the at-risk group of first-degree relatives of AD dementia patients were recruited via public advertisements. In contrast to the HC, who had neither subjective nor objective cognitive impairment, the SCD group presented with complaints of cognitive decline and had to fulfill SCD research criteria [2]. The MCI group consisted of participants with amnestic MCI as defined by an age-, sex- and education-adjusted performance below − 1.5 SD on the delayed recall trial of the Consortium to Establish a Registry for AD (CERAD) word-list episodic memory tests (see also MCI research criteria by [4]). AD dementia participants had to be in a mild dementia stage with Mini-Mental State Examination (MMSE; [34]) performance ≥ 18. Data collection started in 2014, with annual follow-up visits presently ongoing.

The DELCODE study (see **German Clinical Trials Register**: https://drks.de/search/de/trial/DRKS00007966, **registration date 04/05/2015**) complies with the current version of the Declaration of Helsinki. All procedures involving humans were approved by local institutional review boards and ethical committees of all participating sites. All study participants gave written informed consent.

The original study protocol has been subsequently enriched by several supplemental protocols, such as the assessment of LTR. The following descriptions focus on procedures (LTR and MRI) and the study sample relevant to the present analyses.

### LTR paradigm in DELCODE

The DELCODE LTR paradigm has been added subsequently to the study schedule and has been realized by four DELCODE study centers. Hence, first LTR assessments were performed at different follow-up time-points for individual participants. Only the first administration of LTR assessments during the annual study visits was considered for the current analyses (i.e., cross-sectional analyses). Of these LTR assessments (total *N* = 69), 18 were performed at baseline, nine at follow-up 1, 26 at follow-up 2, ten at follow-up 3, and six at follow-up 4.

For the collection of LTR data, participants were asked to recall stimulus material of the following standard neuropsychological tests between 1 and 30 days after their most recent regular study visit: (1) CERAD/ADAS-Cog word list (Consortium to Establish a Registry for AD/AD Assessment Scale-Cognitive Subscale, free recall), and (2) Free and Cued Selective Reminding Task with Immediate Recall (FCSRT-IR, free and cued recall). LTR data were collected during additional study visits or via telephone interview. The retrieval interval varied because many participants returned to the study center for an ancillary DELCODE fMRI study within 30 days of a regular study visit, providing an opportunity for in-person LTR assessment. The current LTR analyses include results of the performance for the delayed recall of the CERAD/ADAS-Cog word list and the FCSRT-IR (free recall). In short, the word list subtest of the CERAD/ADAS-Cog requires encoding and immediate recall of a 10-item word list in three learning trials with a delayed free recall several minutes thereafter. The picture version of the FCSRT-IR begins with a study phase in which participants are asked to search a card containing four pictures (e.g., grapes) for an item that goes with a unique category cue (e.g., fruit). After all four items are identified, immediate cued recall of just those four items is tested, providing retrieval practice while the items are still in working memory. The search is repeated for items not retrieved by cued recall. The search procedure is continued for the next group of four items until all 16 items have been identified and retrieved in immediate recall. The study procedure is followed by three trials of recall, each consisting of free recall followed by cued recall for items not retrieved by recall for a maximum score of 48. FCSRT-IR scores include free recall (FR), cued recall (CR) and total recall (TR = sum of FR and CR). Additionally, each recall trial is separated by a distractor task (counting backwards in steps of three for 20s). Although the relationships between relevant brain structures are not yet fully elucidated, growing evidence suggests that the CERAD/ADAS-Cog word list and the FCSRT-IR tasks rely on hippocampus-dependent memory processes (e.g., [35, 36]), particularly in the context of retrieval deficits ([37–40]). These findings support the use of these cognitive tests to assess hippocampal integrity, with subfields such as the CA1 ( [35, 41]) potentially playing a key role in early cognitive impairment.

The LTR rate as standardized parameter (modified in accordance to [22], modifications only in terms of the tests of interest used here) with values between 0 (complete information loss, possibly indicative of ALF) and 1 (all information recalled during the LTR condition) was defined as follows:

$${\rm{LTR}}\,rate = \frac{{long - term\,retrieval\,\left( {LTR} \right)}}{{last\,trial\,recall\,\left( {trial\:3} \right)}}$$

For example, a subject who had been able to recall eight words in trial 3 of the FCSRT at the regular DELCODE visit, but several days later could only recall two words, had a LTR rate of 0.25. Such, three separate rates as LTR parameters were calculated: (1) LTR rate for the CERAD/ADAS-Cog word list, (2) LTR rate for the FCSRT-IR free recall, and (3) a total LTR rate comprising both subtests (i.e., the mean LTR rate).

### MRI data processing and analyses

**Fig. 1:**
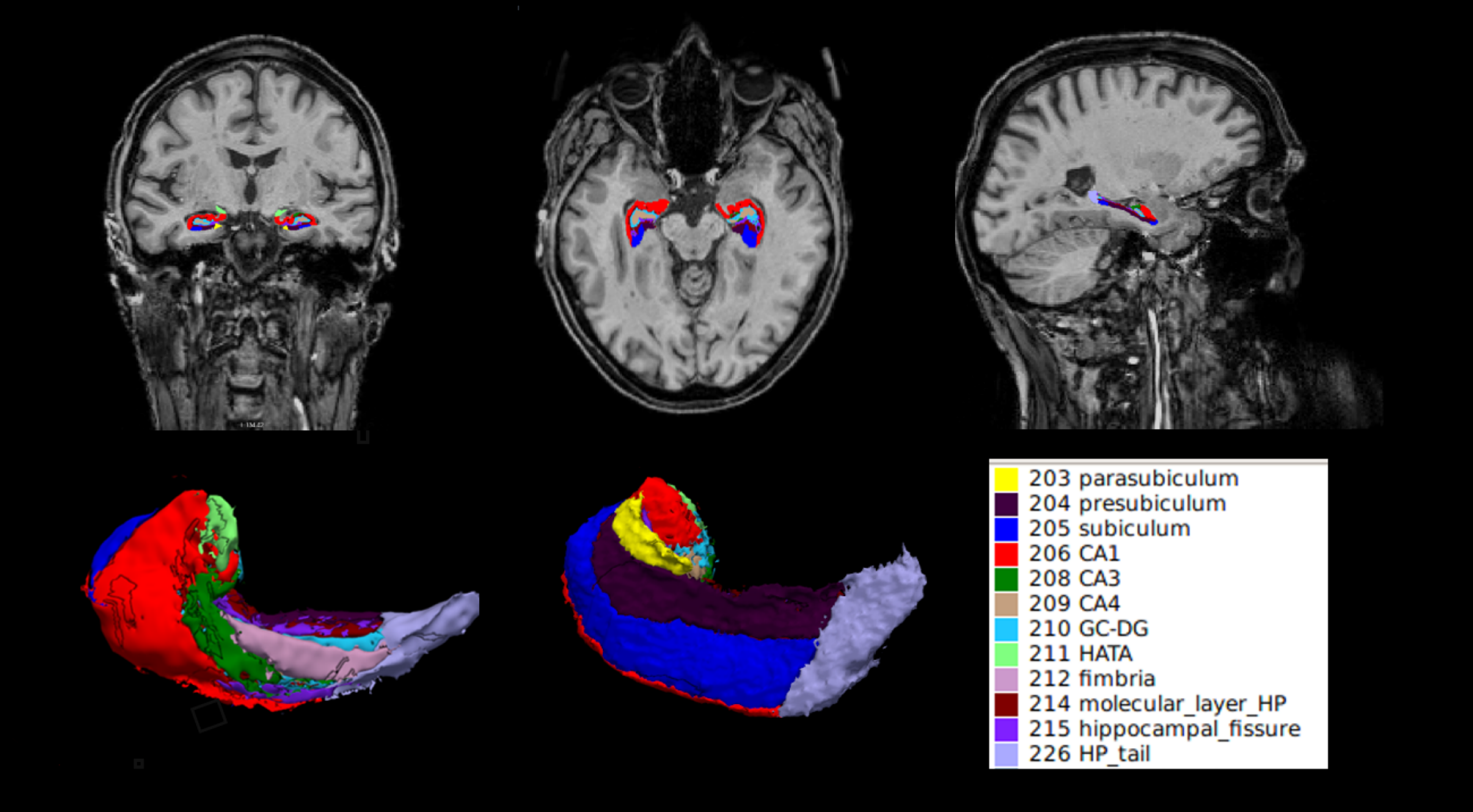
Quality check. Example of a participant’s FreeSurfer segmentation and 3D reconstruction of the hippocampi and constituent subfields

Structural MRI data were acquired as part of the DELCODE study using harmonized protocols across 3T Siemens scanners (Prisma fit, Skyra, TrioTim, and Verio). The imaging protocol included T1-weighted images with 1.0 × 1.0 × 1.0 mm resolution and high-resolution T2-weighted images (0.5 × 0.5 × 1.5 mm) covering the hippocampus. An established method of segmentation of the hippocampus and its corresponding subfields was applied using FreeSurfer v. 6.0 [42–45]. The same method was used in a previous DELCODE study [46] and includes standard preprocessing steps such as brain extraction, B1 bias field correction, tissue segmentation, surface reconstruction, region labeling, and non-linear cortical surface co-registration to a spherical atlas for group comparisons. Whole hippocampi and their constituent subfields have been labeled in each hemisphere: hippocampal tail, subiculum (Sub), CA1, fissure, presubiculum (PreSub), parasubiculum (ParaSub), molecular layer (ML), granule cell layer-molecular layer of the DG, CA3, CA4, fimbria, and hippocampus-amygdala transition area (Hata) region. To ensure that automatic segmentation and corresponding volumes were consistent, manual quality check (QC) was performed using FreeView v2.0 at all stages for all participants in 2D and 3D renderings (Fig. 1) before performing statistical analysis. We did not identify any cases of poorly segmented hippocampi or its respective subfields.

### Final analysis samples

**Fig. 2:**
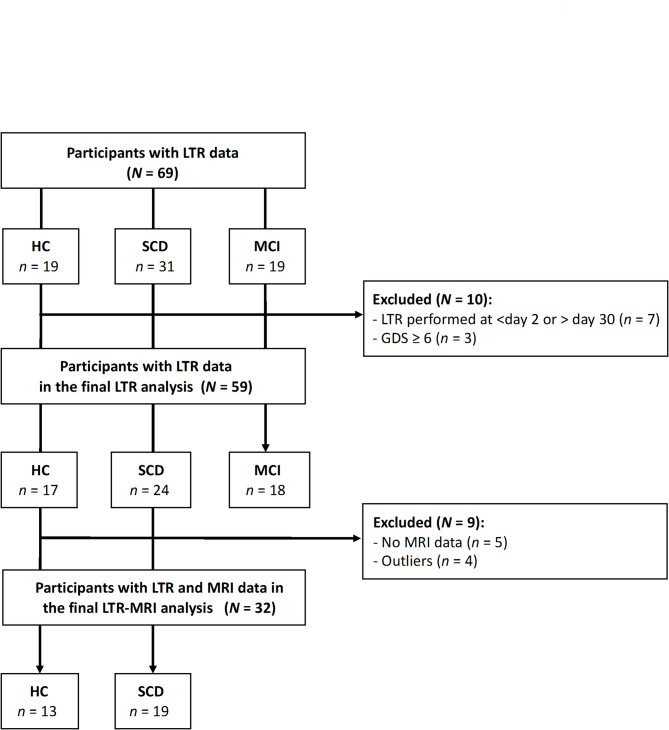
Participants flow. Abbreviations: LTR: long-term retrieval; HC: healthy controls; SCD: subjective cognitive decline; MCI: mild cognitive impairment; GDS: Geriatric Depression scale (15 item-version); MRI: magnetic resonance imaging

Since the current LTR analyses aim at the discrimination of early, preclinical AD stages, HC, SCD and MCI participants with available LTR data of an interim DELCODE data release (October 13, 2022) were included (*N* = 69, Fig. 2). Of those, *n* = 7 participants in which LTR assessment had been performed < day 2 or > day 30 after the respective regular study visit (*n* = 6) or for which the exact LTR assessment date was not given (*n* = 1) were excluded. As clinically relevant depressive symptoms might compromise cognitive performance, *n* = 3 participants with current depressive symptoms– defined by a score of ≥ 6 in the Geriatric Depression Scale (GDS-15 item version; [47, 48])– were also excluded. This resulted in a final LTR analysis sample of *N* = 59 participants (subsamples: *n* = 17 HC, *n* = 24 SCD, *n* = 18 MCI).

LTR-MRI analyses focused on the discrimination of SCD participants and HC. For *n* = 5 participants, no MRI data was available, and *n* = 4 outliers (i.e., ± 2 SD of the mean recall value) were additionally excluded, finally leading to a final LTR-MRI analysis sample of *N* = 32 participants (13 HC, 19 SCD, Fig. 2).

### Statistical analysis

Statistical analyzes were performed in SPSS (IBM Corp. Released in 2022. IBM SPSS Statistics for Windows, Version 29.0. Armonk, NY: IBM Corp). For descriptive analyses of metric variables, we calculated means (*M*) and standard deviations (SD). To evaluate whether groups (HC, SCD, MCI) differed in their LTR rates (CERAD/ADAS-Cog word list, FCSRT-IR free recall, and the average of these two LTR measures) we computed three one-way ANOVAs (between-subjects: three-staged factor). The prerequisite for further variables (e.g., sex, age, MMSE, intelligence, handedness, number of days between recall assessments, intracranial volume) to be considered as covariates in the respective ANOVAs were significant differences between the subgroups. Whenever a significant general difference between all subgroups was observed in the ANOVAs F-test, the possibility of the paradigm to specifically differentiate between the subgroups (HC, SCD, MCI) was assessed with Bonferroni-corrected *post hoc* t-tests within each ANOVA. Furthermore, we calculated ROC curves to assess the degree of SCD vs. HC classification by the paradigm. To assess the degree of a potential relationship between LTR rates and the volume of the CA1 body of each hemisphere, we conducted nonparametric Spearman’s Rho correlations. The initial significance level was set at *p* ≤ 0.05 (two-sided), Bonferroni correction for multiple comparisons was employed as described above.

## Results

### Characteristics of the analysis samples

A total of 59 DELCODE participants were included in the analysis of LTR, belonging to the subgroups HC (*n* = 17), SCD (*n* = 24) and MCI (*n* = 18). At the time-point of LTR assessment participants were *M* = 71.95 years old (*SD* = 5.31). Gender was almost equally distributed (49.6% female, 50.4% male). Education years averaged at 14.02 years (*SD* = 2.62). The average verbal IQ, based on a vocabulary-based intelligence measure (Mehrfachwahl-Wortschatz-Intelligenz-Test; MWT-B; [49]) was *M* = 104.63 IQ (*SD* = 3.2). Cognitive screening with the Mini-Mental Status Examination (MMSE) was indicative of normal performance levels for the complete sample (*M* = 29.08, *SD* = 1.42). The vast majority of participants (93%) were right-handed as measured with the Briggs-Nebes Test of handedness [50]. Days between the regular study visit, including neuropsychological testing, and LTR-assessment averaged at 11.0 (*SD* = 6.4). Participants’ characteristics for each subgroup (HC, SCD, MCI) are summarized in Table 1 and did not reveal any significant differences between these three groups. For LTR-MRI analyses, only HC and SCD participants having LTR and MRI data available were considered, resulting in a smaller subsample of 13 HC and 19 SCD participants. Also, these subsamples did not differ in demographic and clinical characteristics, nor in total hippocampal volumes (see Table 1).

**Table 1:** Study sample: characteristics of participants with LTR data only (*N* = 59) and with LTR and MRI data (*N* = 32)

Participants with LTR data	Total (*N* = 59)	HC (*n* = 17)	SCD (*n* = 24)	MCI (*n* = 18)	F/χ²	*p*
Age (years)	71.19 ± 5.31	69.53 ± 4.63	72.71 ± 5.76	70.72 ± 5.00	1.944	0.153
Gender (% women)^a^	49.6	58.8	37.5	55.6	2.235	0.327
Education (years)	14.02 ± 2.62	14.41 ± 2.09	14.63 ± 2.93	12.83 ± 2.36	2.84	0.067
Premorbid IQ (MWT-B)	104.6 ± 3.20	105.0 ± 2.29	104.9 ± 2.68	103.5 ± 4.27	1.664	0.199
MMSE	29.08 ± 1.42	29.41 ± 0.87	29.21 ± 1.06	28.61 ± 2.06	1.578	0.215
Handedness (% right)^a^	93.0	94.1	91.7	93.8	1.841	0.765
Days between assessments	11.0 ± 6.4	13.0 ± 8.9	10.4 ± 4.7	9.8 ± 5.5	1.251	0.294
Age (years)	70.75 ± 5.41	69.46 ± 4.98	71.63 ± 5.65	-	1.251	0.272
Gender (% women)^a^	40.6	46.2	36.5	-	0.277	0.598
Education (years)	14.36 ± 2.50	14.31 ± 2.02	14.84 ± 2.81	-	0.346	0.561
Premorbid IQ (MWT-B)	105.0 ± 2.54	105.1 ± 2.46	104.8 ± 2.65	-	0.064	0.802
MMSE	29.25 ± 0.98	29.31 ± 0.95	29.21 ± 1.03	-	0.073	0.789
Handedness (% right)^a^	90.6	92.3	89.5	-	0.764	0.683
Days between assessments	11.4 ± 5.8	12.3 ± 7.0	10.8 ± 5.0	-	0.479	0.494
Left hippocampus volume (mm^3^)	3,136.6 ± 291.6	3,147.8 ± 299.9	3,128.9 ± 239.9	-	0.031	0.861
Right hippocampus volume (mm^3^)	3,212.0 ± 281.5	3,188.8 ± 286.5	3,227.9 ± 284.7	-	0.145	0.706

Data presented as means ± standard deviations unless otherwise indicated. P-values refer to comparisons based on univariate analysis of variance or ^a^chi-square tests across groups. LTR: long-term retrieval; HC: healthy controls; SCD: subjective cognitive decline; MCI: mild cognitive impairment; MWT-B: Mehrfachwahl-Wortschatz-Intelligenz-Test (premorbid intelligence measure; [49]); MMSE: Mini-Mental Status Examination. Handedness according to Briggs-Nebes Test [50]

Since no significant between-group differences were detected, none of the variables reported above were further considered as covariates in subsequent analyses.

### Overall and between-group differences (HC, SCD, MCI) in LTR parameters: One-way ANOVA with post hoc tests

**Fig. 3:**
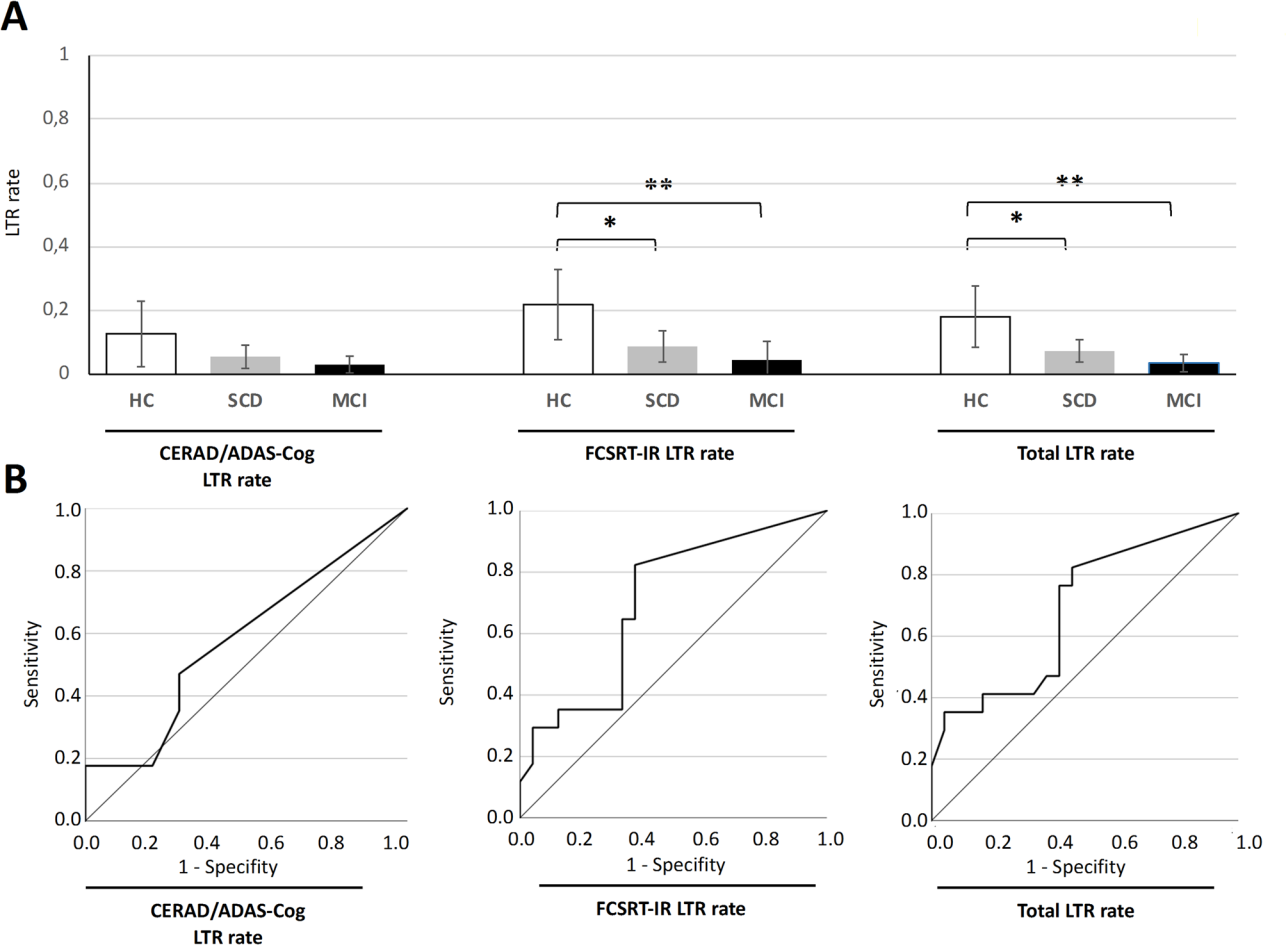
Long-term retrieval (expressed as LTR rates) differs between diagnostic groups HC, SCD, and MCI. (**A**) Results of one-way ANOVA with Bonferroni-adjusted post hoc tests for all diagnostic groups. (**B**) Diagnostic accuracy (discrimination of HC and SCD) by ROC curves. LTR: long-term retrieval; HC: healthy controls; SCD: subjective cognitive decline; MCI: mild cognitive impairment; FCSRT-IR: Free and Cued Selective Reminding Task with Immediate Recall

Descriptive analyses of LTR showed different LTR rates between the subgroups (HC, SCD, MCI), as measured for the (1) CERAD/ADAS-Cog word list, the (2) FCSRT-IR free recall as well as the (3) total LTR rate (combination of these two tests). Numerically, individuals with MCI performed worst for each LTR parameter, followed by participants with SCD, while HC showed the best LTR performance (see Fig. 3A).

Regarding the CERAD/ADAS-Cog word list LTR rates, no significant general difference between groups could be found (*F*(2, 56) = 2.488, *p* = 0.092). At the subgroup level, results point in the same direction as reported descriptively above, but did not reach significance (HC: *M* = 0.127, *SD* = 0.216; SCD: *M* = 0.055, *SD* = 0.091; MCI: *M* = 0.030, *SD* = 0.058; all comparisons *ns*, see Fig. 3A, left panel).

With respect to the FCSRT-IR LTR rates (Fig. 3A, middle panel), a significant general difference between all groups (*F*(2, 56) = 5.451, *p* = 0.007) was found. In Bonferroni-adjusted *post hoc* tests, SCD (*M* = 0.087, *SD* = 0.123) and MCI participants (*M* = 0.045, *SD* = 0.125) showed significantly lower LTR rates than HC (*M* = 0.218, *SD* = 0.232; SCD vs. HC: *p* = 0.040; MCI vs. HC: *p* = 0.008), but no significant difference was determined between SCD and MCI participants (*ns*).

Since the number of retrieved items in each subtest was low (in absolute terms), a total LTR rate (combining the subtests) was computed to increase variance and to reduce potential floor effects. When using the total LTR rate that builds on LTR rates of both single tests, a similar pattern was found as for the FCSRT-IR LTR rate (Fig. 3A, right panel): A statistically significant general difference between HC, SCD and MCI was detected for the LTR parameter total LTR rate (*F*(2, 56) = 6.211, *p* = 0.004). Bonferroni-adjusted *post hoc* t-tests revealed statistically significant lower total LTR rates in SCD (*M* = 0.073, *SD* = 0.089) and MCI (*M* = 0.035, *SD* = 0.059) when compared to HC (*M* = 0.181, *SD* = 0.203; SCD vs. HC: *p* = 0.030; MCI vs. HC: *p* = 0.004), whereas the comparison of the MCI and SCD groups failed to reach significance (*ns*). The results for the total LTR rate therefore reflects primarily the differences that were mainly attributable to differences in the FCSRT-IR LTR rate.

Similar results were obtained when the smaller subgroups of participants who have both LTR and MRI data available were compared (significant differences in FCSRT-IR LTR rates and total LTR rates, but non-significant differences for CERAD/ADAS-Cog word list LTR rates; *data not shown*).

### Exploratory analysis of Raw test data (CERAD/ADAS-Cog word list and FCSRT-IR free recall), including trials preceding LTR assessment: GLM

All LTR rates presented here can be considered low in general, suggesting a high forgetting rate at the LTR assessment compared to the corresponding study visit (see Fig. 3A). For instance, for the CERAD/ADAS-Cog word list recall rate, the majority of MCI, SCD and even HC participants exhibited a LTR rate of 0, reflecting complete forgetting of the study material (MCI: *n* = 14, 77.8%; SCD: *n* = 17, 70.8%; HC: *n* = 9, 52.9%; pairwise comparisons: 0.122 < *p* < 0.731^1^). As this points to a floor effect and may impede the discriminatory value of the LTR paradigm, we computed two additional general linear models (GLMs) for repeated measurements to analyze the group-wise trajectories from the encoding trials to the long-term recall time-point based on the repeatedly measured raw test data (see Figure S1 for the trajectories, Table S1A for detailed results of both GLM and Table S1B for all pairwise comparisons). In both GLMs, all trials and the respective LTR-assessment were included as within-subjects factor, leading to five stages for the CERAD/ADAS-Cog word list (trials 1–3, delayed recall, LTR) and four stages for the FCSRT-IR free recall rate (trials 1–3, LTR). Diagnostic group (MCI, SCD, and HC) were included as three-staged between-subjects factor for both models. Besides main effects (repeated measures, between groups), the interaction effects were analyzed in both models to assess deviations in the trajectories over time between the groups. Overall, both models showed significant between-groups effects (*F*(2, 56) = 21.32 and *F*(2, 56) = 31.71, both *p* < 0.001), and could reliably discriminate between the subgroups (all Bonferroni-corrected pairwise comparisons between subgroups: *p* < 0.029). Moreover, we also observed two significant interaction effects between repeated measures factor and group (*F*(6, 168) = 2.60 and F(8, 224) = 3.85, *p* = 0.020 and < 0.001) which indicated non-parallel trajectories over time: As shown by exploratory pairwise comparisons for each time-point except for the LTR, both, the CERAD/ADAS-Cog word list (a total of 12 *p*-values for assessments before LTR had been assessed (“pre-LTR”), all uncorrected *p* between 0.041 and < 0.001) and the FCSRT-IR free recall (a total of 9 pre-LTR uncorrected *p*values, 7 between 0.002 and < 0.001), could discriminate between HC, SCD and MCI during trials 1–3 (and the (delayed) free recall in CERAD/ADAS-Cog). However, at the last measurement (i.e., LTR), all subgroups showed a major decline in performance, resulting in substantially smaller between-group-differences: For the CERAD/ADAS-Cog word list, no significant differences could be found at all (*p* between 0.054 and 0.205). For the FCSRT-IR free recall, significant differences between HC vs. SCD (*p* = 0.008) and HC vs. MCI (*p* < 0.001) persisted for the LTR time-point, and the difference between SCD vs. MCI was reduced to a non-significant level (*p* = 0.073) if compared to trial 3 (*p* = 0.002). Please see the discussion section for possible implications considering the LTR paradigm.

### HC vs. SCD classification by LTR: ROC-curves

To test the diagnostic utility of LTR in distinguishing HC from SCD, the following analyses focused on classification of HC and SCD participants only. Using ROC analyses, all three LTR parameters (CERAD/ADAS-Cog LTR rate, FCSRT-IR LTR rate total LTR rate) were able to differentiate HC from SCD to different degrees (Fig. 3B). The FCSRT-IR LTR rate had the strongest discriminatory power with an AUC of 0.701 (95% CI 0.537–0.865), indicative of a moderate diagnostic utility (according to [51]), followed by the total LTR rate (AUC = 0.686, 95% CI 0.519–0.854). The smallest AUC was found for CERAD/ADAS-Cog LTR rate (AUC = 0.580, 95% CI 0.399–0.760), corresponding to low discriminatory power / weak diagnostic quality.

### Association of LTR and MRI data in SCD and HC participants: correlational analyses

**Fig. 4:**
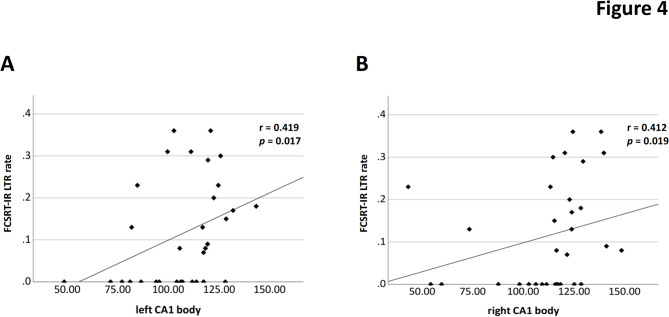
Association of long-term retrieval in FCSRT-IR LTR rate and CA1 volume. (**A**) Left and (**B**) right CA1 body volume were correlated with FCSRT LTR rates in SCD and HC (*N* = 32). Analyses: Spearman-Rho correlations. HC: healthy controls; SCD: subjective cognitive decline; FCSRT-IR: Free and Cued Selective Reminding Task with Immediate Recall

From the ROC curves, the FCSRT-IR LTR rate has shown to discriminate best between HC and SCD participants. MRI analyses were therefore pursued with this LTR parameter only. Using the LTR and MRI data from SCD (*n* = 19) and HC (*n* = 13), we identified a positive correlation between the FCSRT-IR LTR rate and the CA1 body volume in the left (*r* = 0.419, *p* = 0.017, Fig. 4A) and right (*r* = 0.412, *p* = 0.019, Fig. 4B) hemispheres. Furthermore, we found a positive correlation between the FCSRT-IR LTR rate and left CA3 body volume (*r* = 0.525, *p* = 0.002). No other relationship was observed between FCSRT-IR LTR rate and any other subfield or whole hippocampus volumes (all *ns*, see Table S2A). Also, hippocampal volume neither in total nor in subfields significantly differ between HC and SCD (all ns, see Table S2B).

## Discussion

In the context of a growing body of evidence of LTR as a possible readout of ALF in the AD continuum, this study shows that assessing LTR could support the identification of individuals at increased risk for AD, such as older adults with SCD. This distinction by LTR is particularly relevant as conventional clinical assessments of memory and cognition are incapable of differentiating HC and SCD because both groups would, by definition, perform within normal limits. Our findings parallel those of Manes et al. [18] as the first demonstrating impairment in a long-term recall, six weeks after presentation of the material, in older adults with memory complaints and normal cognitive performance (later termed SCD). Two more studies add up to this evidence by showing ALF in SCD 24 h and seven days later [24] or even three months later [21]. Also, in a study by van der Werf et al. [52], SCD participants performed equally to healthy controls in learning and 30-minutes delayed recall and differed only for a 1-week delayed recall. Nevertheless, the authors see only limited value of implementing ALF as standard practice since subjective memory ability ratings and the presence of cognitive dysfunction other than memory dysfunction did not relate to ALF measures. Further research within the early AD continuum also underpins the findings of ALF in cognitively asymptomatic individuals at-risk but differed in terms of defining preclinical stages of AD (e.g., asymptomatic familial AD [22, 23], cognitively asymptomatic ApoE4 carriers [20], and preclinical AD stage 1 [19]). Beyond adding further prove to the findings of ALF or deficient LTR in SCD, our study also aimed at exploring associations of LTR with hippocampus-dependent circuits. Importantly, LTR rates of the FCSRT-IR (free recall), the LTR paradigm with the highest discriminatory power in our sample, correlated with the CA1 body volume– a core hippocampal structure involved memory circuitries of both encoding and retrieval [30, 31, 53, 54].

Measuring LTR and ALF is subject to several methodological challenges, which may have contributed to mixed results in previous research. A major methodological concern pertains to the selection of an appropriate control group, considering many relevant factors such as general cognitive or intellectual status, educational level, and age, all of which should match the experimental group [10]. Fortunately, our analyses revealed no significant differences between diagnostic groups (HC, SCD, MCI) in terms of MMSE results, educational level, premorbid IQ, or age. Our results were therefore unlikely to be biased by differences in such factors. Still, it could be considered critical that only verbal IQ measures were used in determining differences in baseline cognitive status. Thus, we cannot fully exclude that a full-scale IQ would have revealed some between-group differences. Although time intervals between assessments (regular study visit and LTR visit) did numerically vary across groups, those differences were not statistically significant, enabling a reasonable comparison of their LTR.

As recommended previously [10], our study incorporates different stimuli modalities (verbal: CERAD/ADAS-Cog word list; image-based, verbally encoded and recalled: FCSRT-IR) for assessing LTR/ALF. This approach can ascertain whether LTR reflects a more general deficit, or whether LTR is restricted to particular types of information. In the FCST-IR, visual objects are presented, but it predominantly requires verbal naming, verbal categorization and verbal cuing during recall. Interestingly, our results indicate that material of the FCSRT-IR at the LTR assessment, align with the expected patterns: In its free-recall-based LTR rate, HC outperformed the SCD and MCI groups. Furthermore, the FCSRT-IR LTR rate distinguished best between HC and SCD in our sample. One reason for its higher discriminatory precision may originate from its reliance on a deeper, multimodal and semantic encoding process [55, 56]. Previous studies have also reported similar patterns of higher ALF with picture-based material in at-risk groups for developing dementia [18, 22, 25]. A study by Bonner-Jackson et al. also supports the relative sensitivity and higher diagnostic value of visual tasks by finding stronger associations between non-verbal episodic memory measures and hippocampal volumes in a memory clinic population, especially in amnestic MCI participants [57]. In multiple studies, Tort-Merino et al. [19–21] used the Ancient Farming Equipment Task (AFE-T), an associative memory task with free and cued recall as well as recognition memory, for investigating ALF in preclinical AD, asymptomatic ApoE4 carriers, and SCD with amyloid pathology. Since unfamiliar object-pseudoword pairs had to be (over)learnt by individuals with the AFE-T, this paradigm is largely consistent with the verbal elements of the task demands of the FCSRT-IR. We identified only one study in patients with severe traumatic brain injury also employing a 1-week delayed recall of the FCSRT-IR and resulting in an altered learning and long-term consolidation deficit compared to neurologically healthy controls [58]. However, in that study, a word version– instead of the picture version used here– was performed. When considering purely verbal tasks in our study, the LTR rate of the CERAD/ADAS-Cog word list showed a similar pattern as the FCSRT-IR LTR rate results but failed to reach significance in one-way ANOVA and post hoc tests between diagnostic groups with a rather low discriminative power in ROC curve analysis. This is apparently contradictory to previously reported differences in ALF parameters for word list tasks [17, 22, 25]. Yet, it remains unclear for the present study why performance on the CERAD/ADAS-cog word list at the LTR assessment time-point dropped so strongly in all groups and did not differentiate groups consistently. One potential reason might be that compared to more episodic tasks, word-list learning may be more dependent on prefrontal cortex (PFC)-dependent strategy formation during learning. This process is also subject to age-related decline but is less reliant on hippocampal integrity, as previously reported in studies on both genetic and age effects [29, 59].

Although the exploration of both free recall and recognition conditions could provide deeper insights into distinct memory processes potentially impacted in preclinical AD stages, our analysis focused on free recall data. This approach was rooted in the assumption that greater forgetting rates (and thus lower LTR rates) would emerge in free recall conditions [60], resulting in more pronounced decline in SCD compared to HC, thereby increasing discriminatory power. While mitigating ceiling effects, this strategy might, however, have inadvertently contributed to the observed floor effect in LTR values. When analyses for LTR rates on recognition trials of the CERAD/ADAS-cog word list were nevertheless carried out, this indeed helped to overcome this floor effect but would be at the cost of the group-discriminating value of the LTR paradigm (one-way ANOVA for CERAD/ADAS-cog word list recognition LTR rate *ns*, all pairwise comparisons *ns*; *data not shown*). Also, if cued recall conditions for the FCSRT-IR were taken into account (FCSRT-IR total recall, i.e., free + cued recall) for overcoming the floor effect, this would not considerably change the results reported for the FCSRT-IR free recall LTR rates (*data not shown*). Thus, when using the DELCODE LTR paradigm, recognition and cued recall conditions did not provide additional value in discriminating diagnostic groups and when considering both FCSRT-IR LTR rates (free and total recall), these results point to a consolidation and an LTR deficit in SCD and MCI.

Impaired LTR reflects an underlying deficiency in memory processing of specific brain substrates. However, differences in hippocampal total and subfield volume were too subtle to differentiate between HC and SCD and are likely further modulated by other factors not considered here such as amyloid status [61]. We nevertheless did identify a relationship of LTR rate not only with the volume of the CA1 body, as hypothesized and previously reported [30], but also with that of the CA3 body. Within the hippocampus, the CA1 and CA3 regions contribute to distinct aspects of memory processing. CA1, implicated in intermediate- to long-term memory, is associated with the encoding and retrieval of episodic memories [31, 54, 62]. It integrates information from the entorhinal cortex and the CA3 region, facilitating the formation of coherent and context-rich memories [63]. Human lesion studies suggest that CA1 damage can lead to episodic recall deficits [53]. On the other hand, CA3, implicated in short- to intermediate-term memory, is particularly associated with pattern completion and pattern separation [64]. Pattern completion involves retrieving complete episodes from partial cues, crucial for memory recall. Pattern separation refers to distinguishing similar but distinct memory traces. The CA3 region, with its robust connectivity with the dentate gyrus, rapidly encodes novel information [65–67]. Animal studies suggest that disrupting the CA1 region impairs the consolidation and retrieval of long-term memories [53, 68], while CA3 disruption may impair pattern separation and short-term memory [66, 69]. More specifically, with respect to LTR, rodent studies indicate that long-term memories rely on the reactivation of neural patterns within the hippocampus during retrieval, contributing to memory stabilization and consolidation [70]. However, recent findings also point to a role for CA3 in remote memories [71]. Furthermore, we cannot exclude a potential contribution of amyloid pathology to our results, which has been shown to affect hippocampal volumes in SCD [61]. However, since not all participants provided CSF biomaterial for analysis, our sample was too small to robustly assess effects of amyloid status. Future studies using LTR/ALF paradigms should further explore the differentiation between SCD and HC and delve deeper into the mechanisms underlying long-term memory retention and retrieval.

Despite the overall hypothesis-confirming LTR results, several limitations warrant further consideration. First, performance differences between diagnostic groups are likely not restricted to LTR, but have already become apparent in the learning trials/encoding phase, as previously reported in a larger sample from the DELCODE study [72]. By definition, performance differences between HC and SCD participants must, however, have occurred within the normal performance range, as grouping a participant into the SCD category required cognitive performance within normal limits (i.e. test performance of no less than − 1.5 SD below the age, sex, and education-adjusted normal performance on all subtests of the CERAD battery [33]. While the aforementioned subtle differences during learning trials and (early) delayed recall might only questionably help to identify individuals with SCD and at higher-risk for developing dementia [72], the more pronounced differences in LTR may be useful for a more robust distinction between such at-risk individuals and cognitively healthy participants.

Furthermore, it must be noted that all groups, including HC, exhibited a steep performance decline from delayed retrieval at the study visit to the time of LTR assessment, resulting in overall low LTR rates pointing towards a floor effect. In fact, the majority of participants with LTR data did not recall any of the items during LTR, and the remaining participants also recalled only a very small number of items. Importantly, despite a floor effect we still found significant differences between diagnostic groups by LTR as expected. Most notably, and in line with our hypothesis, most participants with complete memory loss (zero-performers) were found in the MCI group, followed by the SCD and then the HC groups. This is to some extent mirrored by the recent observation in a larger sample from DELCODE study that individuals with MCI exhibit substantial disruption of encoding-related brain activity patterns in fMRI, which, on the other hand, showed only gradual differences between HC and SCD [73, 74]. Additionally, previous LTR/ALF studies also reported floor effects [12, 20]. Elliot et al. [10] suggested approaches to overcome biases of ALF estimates by matching initial learning across groups of interest, e.g., by extended exposure times or multiple presentations. These modifications to the original test applications would, however, make data from regular cognitive assessments useless for the categorization of memory performance. Learning to criterion (e.g., at least 80% accuracy) is frequently used in ALF studies to match initial learning, but applying an initial learning criterion for participants to enter LTR analyses to our data leads to a considerable sample size reduction since a sizable proportion of participants (e.g., those with MCI) did not achieve this criterion. Applying this matching procedure nevertheless was also at the cost of failing to reach the significance level for LTR rates (*data not shown*) and such did not improve diagnostic quality of LTR parameters. Furthermore, as stated above, learning to criterion would also prohibit clinical use of standard cognitive tests.

Finally, interval lengths of up to 30 days between the last learning trial and the LTR may have been too long, particularly for individuals at increased dementia risk, and might have contributed to the observed floor effect. The fact that the time interval plays a critical role for retention of learned material dates back to the experiments by Ebbinghaus [9], showing in his famous forgetting curve a lawful relation between retention and time-since-learning. Results are still valid and have been replicated by others (e.g., [75]), with retest intervals up to 31 days. In previous ALF studies using the AFE-T, even retest intervals of three and six months were chosen [19, 20, 21] similarly showing the temporal gradient of memory decay but in an abnormal, accelerated manner (i.e., ALF). However, that paradigm employs overlearning, with participants undergoing two times seven learning sessions on two consecutive days. Interval lengths comparable to the DELCODE LTR design were reported in two studies with ALF assessments (word list, story, and complex visual figure recall) seven days [22] or four weeks [25] after initial learning, but those studies based ALF parameters on performance equated learning through learning to criterion. Future studies should explore a potential benefit from reducing the interval (e.g., to seven days) to optimize LTR assessment while preserving implementation of standardized tests. Potentially, even 24-hour delays might be sufficient to detect subtle performance deficits related to SCD and MCI, as they correlate with both hippocampal volumes [29] and fMRI activation patterns that also distinguish individuals with MCI from healthy controls [73, 76].

### Further strengths and limitations

The DELCODE study from which our sample was drawn is a German-wide, multicenter, observational study conducted in academic memory clinics and comprises deep clinical phenotype data from 1079 participants (HC, SCD, MCI, mild AD dementia, and healthy relatives). Participants undergo assessments at baseline and are followed up during annual visits for a total of 10 years. However, the DELCODE LTR paradigm was introduced subsequently to the original study schedule and has been employed by only a few study centers. Therefore, our analyses could be based only on available data and such small subgroups, which were, however, comparable to group sizes in other studies on ALF in dementia and risk stages [18, 19, 20, 21, 22, 25]. Nevertheless, future studies should consider statistical power issues to finally increase the generalizability of results. An important direction for future research is a potential association of LTR with amyloid pathology, particularly within the SCD group. With CSF biomarker data available in only about 40% of all DELCODE participants, this would have further reduced sample sizes with LTR data considerably. To avoid a further sample size reduction, we thus refrained from conducting more fine-grained subgroup analyses in our present study sample.

The re-assessment of standard memory tests after prolonged retention (i.e., days) as performed in the present LTR paradigm can easily be added to regular cognitive assessments. A similar approach can be applied to both verbal tasks like the Logical memory subtest of the Wechsler memory scale (verbal story recall) [18, 29, 76] or the Verbal Learning and Memory Test (VLMT; word list learning; [76, 77]) as well as tasks using non-verbal abstract visual information like the Rey Complex Figure test [18]. This approach would save time compared to adding specific ALF paradigms to comprehensive standard neuropsychological testing and can even be performed via phone assessments when verbal retrieval tasks (e.g., free recall) are used [76]. More potential approaches to improve the LTR paradigm used here to achieve better diagnostic accuracy have already been discussed above.

In sum, the main limitations of our study, such as small samples, variable retrieval intervals, floor effects, and having no biomarker confirmation of the presence or absence of neurodegeneration, are limitations which are feasible to overcome in future studies.

## Conclusions

With upcoming approval of disease-modifying treatments, early detection of AD gains a new level of importance. With ALF/LTR as an increasingly recognized readout in preclinical AD, its potential utility as an early cognitive marker of subtle cognitive impairment in SCD warrants further investigation– potentially in conjunction with imaging or even new blood-based fluid biomarkers that allow for minimally-invasive and readily available detection of AD pathology [78, 79]. Considering that memory network integrity shows measurable disruption in MCI, but is still relatively preserved in SCD [73, 74], early detection of high-risk individuals at the pre-MCI stage with affordable means like ALF/LTR tests, combined with imaging and/or blood biomarkers, may help to pave the way for early intervention in this group.

If LTR measurement is designed economically as an add-up to standard cognitive tests, it bears the potential to improve diagnostic quality in at-risk individuals. Further research should focus on optimizing the assessment of LTR for clinical implementation by establishing an appropriate time interval, as well as cut-offs and norms. Thereby, future clinical trials and intervention strategies can include patients at risk who, as of today, are still indistinguishable from HC in the conventional diagnostic workup.

## Electronic Supplementary Material

Below is the link to the electronic supplementary material.

Supplementary Material 1

## Data Availability

The data supporting the findings of this study are available on reasonable request from the corresponding author. The data are not publicly available due to privacy or ethical restrictions.

## Footnotes

1 Multiple Χ^2^-squared tests/Fisher’s exact tests for dichotomized recall rate: (1) zero performers (LTR rate = 0) vs. performers (LTR rate > 0).
