# Supplementary material for: Long-term retrieval performance is associated with CA1 hippocampal volume in older adults and individuals at risk for dementia
Source: Alzheimers Res Ther. 2025 Aug 22;17:195. doi: 10.1186/s13195-025-01833-4 (PMC12372344; doi:10.1186/s13195-025-01833-4)
Supplement: Supplementary file 1 — Supplementary Material 1 [file 13195_2025_1833_MOESM1_ESM.doc]

**Supplementary Material**

**Table S1A.** General linear models for repeated measurement (GLM) for CERAD/ADAS-Cog word list recall and FCSRT-IR free recall (raw test data)

|  | **Repeated measures**  **(*F*, *p*, η^2^)** | **Between groups**  **(*F*, *p*, η^2^)** | **Interaction**  **(*F*, *p*, η^2^)** |
| --- | --- | --- | --- |
| CERAD/ADAS-Cog word list | *F*(4, 224) = 506.64, ***p* < .001**, η^2^ = 0.90 | *F*(1, 56) = 21.32, ***p* < .001**, η^2^ = 0.43 | *F*(8, 224) = 3.85, ***p* < .001**, η^2^ = 0.12 |
| FCSRT-IR free recall | *F*(3, 168) = 278.25, ***p* < .001**, η^2^ = 0.83 | *F*(1, 56) = 31.71, ***p* < .001**, η^2^ = 0.53 | *F*(6, 168) = 2.60, ***p* = .020**, η^2^ = 0.09 |

*Notes. GLM*: *F*-/*p*-/ η2-values for repeated-measures- between groups- and interaction-effect

**Table S1B.** Pairwise comparisons for CERAD/ADAS-Cog word list recall and FCSRT-IR free recall (raw test data)

| **Cognition scores** |  | **Trial 1** | **Trial 2** | **Trial 3** | **Delayed recall** | **LTR** |
| --- | --- | --- | --- | --- | --- | --- |
| CERAD/ADAS-Cog word list | HC vs. SCD | *t*(39) = 2.12, ***p* = .039** | *t*(39) = 2.73, ***p* = .009** | *t*(39) = 3.13, ***p* = .003** | *t*(39) = 3.13, ***p* = .003** | *t*(39) = 1.50, *p* = .141 |
|  | HC vs. MCI | *t*(33) = 4.83, ***p* < .001** | *t*(33) = 5.07, ***p* < .001** | *t*(33) = 5.54, ***p* < .001** | *t*(33) = 5.65, ***p* < .001** | *t*(33) = 2.00, *p* = .054 |
|  | SCD vs. MCI | *t*(40) = 2.90, ***p* = .006** | *t*(40) = 3.38, ***p* = .002** | *t*(40) = 2.11, ***p* = .041** | *t*(40) = 3.64, ***p* = .001** | *t*(40) = 1.29, *p* = .205 |
| FCSRT-IR free recall | HC vs. SCD | *t*(39) = 4.99, ***p* < .001** | *t*(39) = 4.41, ***p* < .001** | *t*(39) = 5.93, ***p* < .001** | – | *t*(39) = 2.80, ***p* = .008** |
|  | HC vs. MCI | *t*(33) = 5.94, ***p* < .001** | *t*(33) = 4.85, ***p* < .001** | *t*(33) = 7.27, ***p* < .001** | – | *t*(33) = 3.33, ***p* = .002** |
|  | SCD vs. MCI | *t*(40) = 1.24, *p* = .221 | *t*(40) = 1.63, *p* = .111 | *t*(40) = 3.23, ***p* = .002** | – | *t*(40) = 1.84, *p* = .073 |

*Notes.* Captions: t-tests with *p-*values Cohens’s *d*. LTR (long-term retrieval) refers to the long-term recall of stimuli learned before (i.e. free recall after > 1 and ≤ 30 days after the last regular study visit).


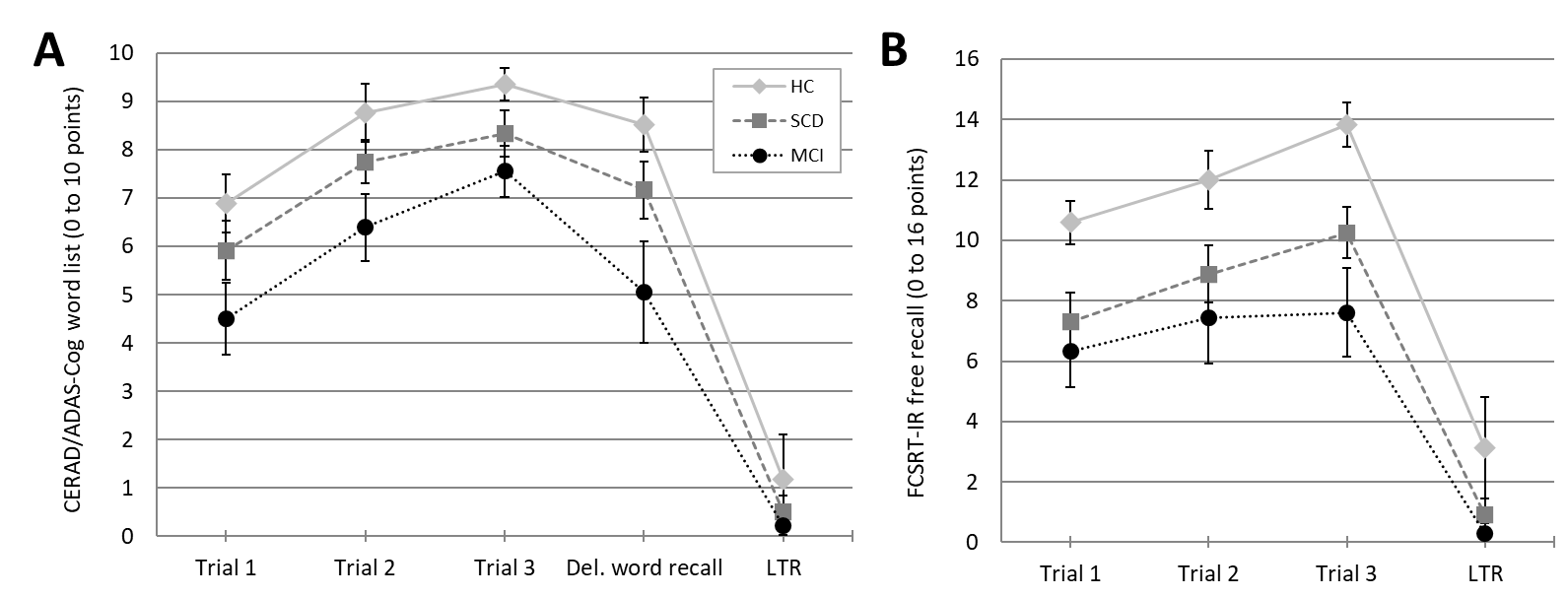


**Figure S1. Trajectories of recall rates (raw data).** Mean values with 95% confidence intervals, differentiated by subgroups HC (healthy controls, *n* = 17), SCD (subjective cognitive decline, *n* = 24), and MCI (mild cognitive impairment, *n* = 18). **(A)** CERAD/ADAS-Cog word list. **(B)** FCSRT-IR free recall. Please see **Tables S1** for all pairwise comparisons and main effects (GLM).

**Table S2A.**

Spearmen-Rho correlations of the LTR rate of the FCSRT-IR (free recall) and hippocampal subfields

|  | **LTR rate FCSRT-IR** | |  | **LTR rate FCSRT-IR** | |
| --- | --- | --- | --- | --- | --- |
| **Hippocampal subfields (left)** | **r** | ***p*** | **Hippocampal subfields (right)** | **r** | ***p*** |
| Whole hippocampus | 0.014 | *0.938* | Whole hippocampus | 0.096 | *0.630* |
| Hippocampal tail | 0.210 | *0.248* | Hippocampal tail | 0.203 | *0.265* |
| Subiculum body | -0.065 | *0.724* | Subiculum body | -0.052 | *0.777* |
| **CA1 body** | **0.419** | ***0.017**** | **CA1 body** | **0.412** | ***0.019**** |
| Subiculum head | -0.240 | *0.185* | Subiculum head | -0.037 | *0.839* |
| Hippocampal fissure | 0.119 | *0.515* | Hippocampal fissure | -0.076 | *0.678* |
| Presubiculum head | -0.167 | *0.361* | Presubiculum head | -0.063 | *0.733* |
| CA1 head | 0.029 | *0.874* | CA1 head | 0.019 | *0.920* |
| Presubiculum body | -0.275 | 0.128 | Presubiculum body | *-0.290* | *0.108* |
| Parasubiculum | -0.121 | 0.509 | Parasubiculum | *0.270* | *0.135* |
| ML HC head | -0.004 | 0.983 | ML HC head | *-0.010* | *0.956* |
| ML HC body | -0.107 | 0.561 | ML HC body | *-0.039* | *0.833* |
| GC ML DG head | 0.143 | 0.435 | GC ML DG head | *0.130* | *0.479* |
| **CA3 body** | **0.525** | ***0.002***** | CA3 body | 0.324 | *0.070* |
| GC ML DG body | -0,116 | 0.528 | GC ML DG body | *0.009* | *0.963* |
| CA4 head | -0.019 | 0.916 | CA4 head | *0.061* | *0.740* |
| CA4 body | 0.093 | 0.613 | CA4 body | *0.047* | *0.798* |
| Fimbria | 0.043 | 0.816 | Fimbria | *0.026* | *0.887* |
| CA3 head | 0.149 | 0.416 | CA3 head | *0.080* | *0.662* |
| HATA | 0.055 | 0.766 | HATA | *0.155* | *0.398* |
| Hippocampal body | 0.007 | 0.970 | Hippocampal body | *0.056* | *0.763* |
| Hippocampal head | -0.050 | 0.784 | Hippocampal head | 0.010 | 0.957 |

*Notes. *p ≤ 0.05; **p ≤ 0.01. Abbreviations:* FCSRT-IR: Free and Cued Selective Reminding Task with Immediate Recall; ML: molecular layer; HC: hippocampus; GC: granule cell; DG: dentate gyrus; HATA: hippocampus-amygdala-transition-area.

**Table S2B.**

Mann-Whitney-U-tests for differences between healthy controls (HC) and participants with subjective cognitive decline (SCD) in hippocampal subfields

| **Hippocampal subfields (left) in mm³** | **HC**  (n = 13) | **SCD**  (n = 19) | ***p*** | **Hippocampal subfields (right) in mm³** | **HC**  (n = 13) | **SCD**  (n = 19) | ***p*** |
| --- | --- | --- | --- | --- | --- | --- | --- |
| Whole hippocampus | 3148 ± 300 | 3129 ± 294 | *0.677* | Whole hippocampus | 3189 ± 286 | 3228 ± 285 | *1.000* |
| Hippocampal tail | 496 ± 70 | 476 ± 74 | *0.495* | Hippocampal tail | 532 ± 61 | 501 ± 78 | *0.238* |
| Subiculum body | 241 ± 31 | 230 ± 23 | *0.117* | Subiculum body | 236 ± 29 | 233 ± 26 | *0.448* |
| CA1 body | 110 ± 16 | 105 ± 23 | *0.734* | CA1 body | 119 ± 21 | 108 ± 27 | *0.238* |
| Subiculum head | 202 ± 29 | 184 ± 32 | *0.136* | Subiculum head | 192 ± 30 | 194 ± 33 | *0.821* |
| Hippocampal fissure | 174 ± 28 | 171 ± 32 | *0.520* | Hippocampal fissure | 184 ± 28 | 185 ± 27 | *1.000* |
| Presubiculum head | 138 ± 28 | 129 ± 24 | *0.570* | Presubiculum head | 128 ± 19 | 130 ± 21 | *0.762* |
| CA1 head | 502 ± 44 | 493 ± 63 | *0.448* | CA1 head | 501 ± 62 | 534 ± 69 | *0.305* |
| Presubiculum body | 170 ± 28 | 166 ± 37 | *0.404* | Presubiculum body | 150 ± 29 | 155 ± 34 | *0.650* |
| Parasubiculum | 68 ± 18 | 69 ± 20 | *0.910* | Parasubiculum | 67 ± 12 | 65 ± 14 | *0.734* |
| ML HC head | 276 ± 34 | 290 ± 53 | *0.762* | ML HC head | 251 ± 28 | 273 ± 51 | *0.126* |
| ML HC body | 182 ± 17 | 196 ± 26 | *0.195* | ML HC body | 212 ± 37 | 214 ± 39 | *0.623* |
| GC ML DG head | 138 ± 15 | 141 ± 18 | *0.791* | GC ML DG head | 144 ± 20 | 149 ± 28 | *0.821* |
| CA3 body | 76 ± 10 | 77 ± 12 | *0.734* | CA3 body | 82 ± 14 | 80 ± 15 | *0.791* |
| GC ML DG body | 114 ± 13 | 118 ± 17 | *0.762* | GC ML DG body | 117 ± 15 | 121 ± 13 | *0.623* |
| CA4 head | 114 ± 13 | 118 ± 14 | *0.182* | CA4 head | 121 ± 15 | 124 ± 21 | *0.880* |
| CA4 body | 103 ± 10 | 104 ± 10 | *0.762* | CA4 body | 108 ± 11 | 107 ± 10 | *0.910* |
| Fimbria | 58 ± 20 | 66 ± 23 | *0.305* | Fimbria | 58 ± 16 | 65 ± 16 | *0.182* |
| CA3 head | 108 ± 22 | 112 ± 20 | *0.677* | CA3 head | 117 ± 23 | 119 ± 23 | *0.910* |
| HATA | 52 ± 9 | 55 ± 13 | *0.623* | HATA | 56 ± 10 | 56 ± 13 | *0.596* |
| Hippocampal body | 1055 ± 102 | 1061 ± 83 | *0.880* | Hippocampal body | 1082 ± 121 | 1083 ± 88 | *0.791* |
| Hippocampal head | 1597 ± 155 | 1592 ± 203 | *0.734* | Hippocampal head | 1575 ± 153 | 1643 ± 209 | *0.472* |

*Notes:* Means ± standard deviations presented. **p ≤ 0.05; **p ≤ 0.01. Abbreviations:* ML: molecular layer; HC: hippocampus; GC: granule cell; DG: dentate gyrus; HATA: hippocampus-amygdala-transition-area.
